# Identifying clinical and lifestyle factor mediators of the association between socioeconomic status and colorectal cancer mortality

**DOI:** 10.1016/j.ssmph.2026.101952

**Published:** 2026-07-18

**Authors:** Thomas P. Lawler, Oluwatoyosi Ogunmuyiwa, Rida A. Khatri, Mark D. Steinwandel, Ronald E. Gangnon, Martha J. Shrubsole, Shaneda Warren Andersen

**Affiliations:** a University of Wisconsin Carbone Cancer Center, Madison, WI, 53726, USA; b Department of Population Health Sciences, School of Medicine and Public Health, University of Wisconsin-Madison, 610 Walnut St, WARF Office Building, Madison, WI, 53726, USA; c International Epidemiology Field Station, Vanderbilt Institute for Clinical and Translational Research, 1455 Research Blvd, Suite 550, Rockville, MD, 20850, USA; d Department of Biostatistics and Medical Informatics, School of Medicine and Public Health, University of Wisconsin-Madison, Madison, WI, 53726, USA; e Division of Epidemiology, Department of Medicine, Vanderbilt Epidemiology Center, Vanderbilt-Ingram Cancer Center, Vanderbilt University School of Medicine, 2525 West End Avenue, Nashville, TN, 37203-1738, USA

**Keywords:** Colorectal neoplasms, Socioeconomic disparities in health, Healthcare disparities, Healthy lifestyle, Survival, Mortality

## Abstract

**Purpose:**

Higher SES is associated with lower mortality among colorectal cancer (CRC) survivors. We performed a mediation analysis to identify factors that mediate the association between household income and CRC mortality.

**Methods:**

Data arise from 1038 participants of the prospective Southern Community Cohort Study (SCCS) who developed incident CRC after enrollment (2002-2009); 73% self-identify as Black. Fifty-nine percent self-reported household income <$15,000/year. Mortality outcomes were determined via the National Death Index. Proportional hazards models were used to estimate hazard ratios (HRs) with 95% confidence intervals (CIs) for mortality by income level. Mediation analysis was used to estimate indirect effects of stage, healthcare-access and use, comorbidities and health behaviors on mortality.

**Results:**

Higher income was associated with lower risk for all-cause mortality (≥$15,000 vs. <$15,000/year: HR = 0.70, 95%CI:0.58-0.84) and CRC-specific mortality (HR = 0.75, 95%CI:0.59-0.94). Differences in stage and surgical resection together explained 33% of the association with CRC-specific mortality and 16% for all-cause mortality. Smoking history explained 11% of the association with all-cause mortality and 9% for CRC-specific mortality. Diet explained 9% of the association with each outcome. Adjustment for all potential mediators explained 26% and 44% of the associations with all-cause and CRC-specific mortality, respectively. Associations between income and mortality were similar for participants with local/regional tumors only.

**Conclusions:**

Differences in early detection and surgical resection are the strongest mediators of the association between income and mortality. SES-targeted health policies and patient navigation will improve CRC outcomes. Additionally, health policies to address smoking cessation and food availability will create opportunities for interventions to promote health equity.

## Highlights

• Higher income is associated with lower colorectal cancer mortality risk.
• Diagnosis stage and surgical resection together explained 33% of the survival gap.
• Differences in smoking history and diet pattern accounted for roughly 10% each.
• Insurance coverage had minimal impact on outcomes.
• Additional resources beyond health insurance are needed to improve cancer outcomes.

## 1 Introduction

Colorectal cancer (CRC) is the 2nd leading cause of cancer mortality in the United States (US), and it is predicted that CRC will be responsible for more than 55,000 deaths for 2026 (Siegel et al., 2026). Notably, the burden of CRC mortality is not distributed equally where longstanding socioeconomic status (SES) disparities exist (Siegel et al., 2015). Household income and education are the most studied proxies for SES and have been inversely associated with mortality after a CRC diagnosis (Aloysius et al., 2021; Cheng et al., 2021; Gabriel et al., 2018; Holowatyj et al., 2025; Zhu et al., 2023).

Socioeconomic disparities in CRC mortality may reflect differences in healthcare access and screening by SES. Individuals with lower SES are less likely to have adequate insurance coverage or a regular healthcare provider, leading to lower rates of preventive screening and advanced stage at diagnosis (Carethers & Doubeni, 2020). Individuals with lower SES are less likely to receive guideline-concordant care (Herb et al., 2022). Specifically, among individuals diagnosed with CRC, those with lower SES are less likely to undergo surgical resection of the primary tumor site in accordance with expert guidelines (Akinyemiju et al., 2018). Consequently, disparities in healthcare-related factors may contribute to unequal CRC mortality outcomes.

Disparities are further exacerbated by differences in health behaviors and comorbidities (Fang et al., 2023; Zhang et al., 2021). Nationally-representative surveys report associations between lower SES status and a higher prevalence of harmful health behaviors and comorbidities (Fang et al., 2023; Zhang et al., 2021) linked to CRC mortality, including current smoking (Liang et al., 2009; Ordonez-Mena et al., 2017; Walter et al., 2015), unhealthy diet (Castro-Espin & Agudo, 2022), inadequate physical activity (Choy et al., 2022), and type 2 diabetes (Lawler et al., 2024; Ling et al., 2021). Individuals who perform more healthy behaviors may reduce mortality risk by lessening the likelihood and influence of comorbidities while also alleviating chronic inflammation, which is linked to tumor progression and metastasis (Hibino et al., 2021). Healthy lifestyle can also reduce the risk for visceral adiposity, metabolic syndrome, and type 2 diabetes, which are associated with poor CRC prognosis (Lu et al., 2023). Individuals with lower SES may face additional challenges to follow health behavior guidelines due to financial constraints, limited availability of healthful resources in low-income areas (Larson et al., 2009), and the effects of chronic stress (Sinha, 2008). While lifestyle risk factors and comorbidities may directly impact mortality outcomes, there is growing awareness that cancer risk factors can also impact the invasiveness and molecular characteristics of developing tumors, with potential implications for treatment and mortality (Ogino et al., 2026). The prospective-cohort incident-tumor biobank method (PCIBM) is an emerging approach to develop tumor biobanks in longitudinal research, with a goal to understand how modifiable risk factors impact tumor molecular characteristics (Ogino et al., 2026).

The prospective Southern Community Cohort Study (SCCS) is a unique resource with the strength of enrolling a cohort with a wide distribution of socioeconomic positions and experiences. The current study fits within the PCIBM framework for cancer research, including detailed information about tumor pathobiological characteristics that impact mortality, such as stage. Cohort data finds lower household income is associated with higher risk for mortality after an incident CRC diagnosis (Holowatyj et al., 2025). However, there are currently no studies that have examined the potentially modifiable factors that mediate the protective association between higher individual income and better survival among individuals with CRC. Such studies are necessary to identify and prioritize clinical and population level interventions to reduce inequalities. Herein, we expand upon the protective finding between income and mortality by examining and identifying healthcare- and lifestyle-related factors which mediate the association between SES and mortality after CRC diagnosis. Identifying factors that mediate the association between SES and mortality among people with CRC supports the development of interventions to extend survival and promote health equity.

## 2 Fundamental Causes Theory

This project builds on Fundamental Causes Theory, developed by Link and Phelan (1995), a framework that aims to identify root causes of socioeconomic disparities for the purposes of supporting interventions and policy decisions to promote health equity. Fundamental Causes Theory has previously been used as a framework for investigating socioeconomic disparities in CRC mortality (Saldana-Ruiz et al., 2013) and posits that disparities arise and persist over time because individuals with higher SES employ their greater resources to adapt health-promoting behaviors and access necessary treatments (Link & Phelan, 1995). Relevant resources may may include income, wealth, knowledge, and access to insurance and other healthcare resources, including preventive screening and curative treatments. In the context of CRC, socioeconomic status may act as a fundamental cause of disparities when high-SES individuals utilize their resources to access health insurance, preventive endoscopy screening, or surgical resection at higher rates compared to low-SES individuals (Carethers & Doubeni, 2020). Consequently, differences in resources would be expected to result in higher rates of early cancer detection for high-SES individuals with more favorable surgical outcomes. High-SES individuals may also utilize their greater knowledge or financial resource to better adhere to lifestyle guidelines linked to better prognosis, including for overall diet pattern (e.g. the Dietary Guidelines for Americans), avoiding smoking, and limiting alcohol consumption (Phelan et al., 2010). Previously, Fundamental Causes Theory was employed to investigate how differences in screening rates and behavioral risk factors contribute to socioeconomic disparities in area-level CRC mortality rates (Clouston et al., 2020). However, the extent to which modifiable factors mediate the impact of SES on mortality risk for individuals with CRC is currently unknown. Using the Fundamental Causes Theory framework, we have performed a mediation analysis to investigate the extent to which socioeconomic disparities in CRC mortality are explained by healthcare-related factors (e.g. diagnosis stage, surgery), lifestyle, and comorbidities. The present sample is predominantly composed of individuals with much lower SES than is typical in epidemiological studies. Consequently, we assess whether relatively modest differences in income are associated with improved outcomes for people with CRC, as well as the potentially modifiable risk factors that may mediate the association.

## 3 Materials and methods

### 3.1 Study design

In this prospective cohort study, data arise from 1038 participants who developed incident CRC after enrollment. Between 2002 and 2009, approximately 85,000 English-speaking participants were enrolled in the SCCS aged 40-79 years from 12 states in the southeastern US. Eighty-six percent were enrolled from community health centers, while the remaining 14% were recruited via general population mailing and telephone calls. Details concerning SCCS study design and recruitment were published previously (Signorello et al., 2005). At enrollment, participants completed detailed written questionnaires. Questionnaires included self-reported assessment of biological sex (male, female), self-identified race and ethnicity (White, Black/African American, Hispanic/Latino, Asian or Pacific Islander, American Indian or Alaska native, or other racial or ethnic group), annual household income, education, marital status, medical history, current medications, family history of cancer, cancer screening history, lifestyle risk factors including smoking history and alcohol consumption, physical activity, and diet. Follow-up occurred via mailed questionnaires at approximately 5-year intervals, with the third follow-up completed in 2018. All questionnaires utilized for the present study were developed by the SCCS and have been published online (Southern Community Cohort Study, n.d.). All participants provided written informed consent. The Institutional Review Boards of the Vanderbilt University Medical Center and Meharry Medical College provided approval for this study. All study activities conformed to the tenets of the Declaration of Helsinki.

### 3.2 Inclusion and exclusion criteria

The inclusion criterion was incident CRC after study enrollment (N = 1212). The exclusion criteria were self-reported history of cancer at enrollment (excluding non-melanoma skin cancer, N = 105), missing data concerning annual household income (N = 42), and diagnosis of CRC *in situ* (stage 0, N = 27). In total, 1038 participants were included in the analysis.

### 3.3 Measurement of annual household income

Annual household income was assessed at study enrollment via questionnaire using the following categories: <$15,000; $15,000-24,999; $25,000-49,999; $50,000-74,999; $75,000-99,999; ≥$100,000.

### 3.4 Assessment of incident colorectal cancer and mortality outcomes

Participants with incident CRC were identified via linkage to state cancer registries and the National Death Index (NDI). Cancer of the colon or rectum was defined as International Classification of Diseases for Oncology-3 codes C180-C189, C199, and C209. Linkage to state cancer registries was complete through 2016-2019 depending on the registry. Mortality outcomes from the NDI included overall mortality and CRC-specific mortality. Linkage to the NDI was complete through December 31, 2020.

### 3.5 Measurement of clinical risk factors for mortality

The Surveillance, Epidemiology, and End Results (SEER) summary stage was obtained from state cancer registries. Likewise, whether a participant completed surgical resection of the primary tumor during the first course of treatment was determined from the state registries. Insurance coverage at enrollment (yes, no) was assessed via self-report. History of CRC screening (yes, no) was assessed via self-report and defined as ever completion of colonoscopy, sigmoidoscopy, or fecal occult blood testing reported at enrollment or on any follow-up questionnaire completed prior to CRC diagnosis.

### 3.6 Measurement of lifestyle risk factors for mortality

Smoking status (never, former, current) and total pack years smoked were self-reported at enrollment. For alcohol consumption, participants reported the average number of drinks per week over the past year, including beer, wine, and liquor. Participants were then categorized as non-drinkers (0 drinks/day), light/moderate drinkers (<1 drink/day for women, <2 drinks/day for men), or heavy drinkers (≥1 drink/day for women, ≥2 drinks/day for men). Participants also self-reported the total duration of moderate and vigorous physical activity completed each week, and participants who reported completing ≥75 min/week of vigorous activity or ≥150 min/week of combined moderate or vigorous activity were classified as meeting guidelines (Piercy et al., 2018). At enrollment, participants completed a food-frequency questionnaire with 89 items across 12 categories, as previously described (Buchowski et al., 2003) Adherence to the Dietary Guidelines for Americans (2010) was assessed via calculation of the Healthy Eating Index score, as previously described (Guenther et al., 2013) Participants self-reported doctor's diagnosis of diabetes (yes/no) and age at diabetes diagnosis at enrollment and on all subsequent follow-up questionnaires. Diabetes status (yes/no) was defined as self-reported doctor's diagnosis of diabetes on at least one questionnaire completed prior to CRC diagnosis. Total duration of diabetes was calculated as the number of years between the age at diabetes diagnosis and the age at CRC diagnosis and categorized as follows: no diabetes; diabetes <5 years; diabetes ≥5 and < 10 years; and diabetes ≥10 years. Participants whose diabetes was diagnosed after the CRC diagnosis were classified as not having diabetes (N = 30).

### 3.7 Statistical analysis

Participant demographic, clinical, and lifestyle characteristics were compared by annual household income at enrollment (≥$15,000 vs. <$15,000) and overall mortality status at the end of follow-up. The association between income and all covariates was assessed via logistic regression with adjustment for age at enrollment, sex, race, enrollment source, and marital status. The association between household income and mortality was assessed via Cox proportional hazards models and described using hazard ratios (HRs) with 95% confidence intervals (CIs). Follow-up began at the month of incident CRC diagnosis and ended at the month of death or was censored at the end of follow-up. For participants with a follow-up time of zero months (i.e. age at CRC diagnosis and mortality were equivalent), one month was added to the mortality age. The proportional hazards assumption was assessed via log-log survival curves for all covariates and was considered met. For most covariates, data were missing for approximately 1-2% of observations. Consequently, missing values were imputed using the sex- and race-specific mode (categorical variables) or median (continuous variables). Missing values were more common for tumor stage (22%) and surgery status (18%), and a separate category was used to indicate missingness for these variables. Model 1 was adjusted for age (as the time scale), sex (male, female), race (Black, White, Other), enrollment source (community health center, mail/telephone), and marital status (married/living with partner, separated/divorced, widowed, single/never married). Marital status was included in model 1 to account for the potential impact of multiple incomes on total household income. The association between income and mortality outcomes was investigated in the full sample and stratified by race (Black, White), sex (male, female), and education (<high school graduate, high school graduation or above). A statistical test for interaction was performed by adding an interaction term to model 1 for each potential effect modifier.

To determine whether the association between household income and mortality is influenced by healthcare- and lifestyle-related factors, we performed a mediation analysis by adding the following variables to model 1: diagnosis stage (local, regional, distant, missing); CRC screening (no, yes); insurance status (no, yes); surgical resection (no, yes, missing); smoking history (defined using cross-classification of smoking status (never, former, current) and quintiles of pack-years smoked); meeting physical activity guidelines (yes, no), alcohol consumption (non-drinker, light/moderate, heavy), Healthy Eating Index score (quintiles); and diabetes duration at CRC diagnosis (no diabetes; diabetes <5 years; diabetes ≥5 and < 10 years; and diabetes ≥10 years). The indirect effect (i.e. the proportion of the association between income and mortality attributable to the mediator(s)) was calculated as follows: $\left(\beta_{1}-\beta_{0}\right)∕\left(1-\beta_{0}\right)\times 100$, where β_0_ represents the logHR for income in model 1 and β_1_ represents the logHR for income in the model including the potential mediating variable. Mediation analysis was performed by adding each potential mediator to model 1 individually. However, given the strong relationship between stage and completion of surgery, stage and surgery were added to model 1 collectively. Further, a cumulative mediation analysis was performed by adding the following sets of mediators to model 1: 1) stage and surgery only; 2) stage and healthcare variables (surgery, stage, screening, and insurance); and 3) all potential healthcare- and lifestyle-related mediators. Because participants with distant stage tumors may not be eligible to receive surgical resection of the primary tumor site, a sensitivity analysis was done limited to participants with local or regional tumors only. All p-values are two-sided, and statistical significance was defined as p-value <0.05. All analyses were performed using SAS version 9.4 (*SAS Inc., Cary, NC*) in the year 2024.

## 4 Results

In total, 1038 participants who developed incident CRC after SCCS enrollment were included in the analysis. The median (IQR) age at CRC diagnosis was 61 (56-68) years, and the median (IQR) time between enrollment and CRC diagnosis was 7.3 (4.0-10.8) years. Most participants self-reported female sex (58%) and Black racial identity (73%) (Table 1). There were 27 participants who self-reported non-Black, non-White racial identity (Hispanic/Latino [7], Asian or Pacific Islander [1], American Indian or Alaska native [1], Other [1], mixed race [17]). At the end of follow-up, 58% of participants were deceased (N = 606) and 35% died from CRC (N = 364). The median time between CRC diagnosis and death or censoring was 3.1 years (IQR: 0.7-7.7 years, income < $15,000/year: 2.5 years, income ≥ $15,000/year: 4.1 years). Higher overall mortality was associated with enrollment via community health center, male sex, self-identified Black race, lower education, non-married status, advanced diagnosis stage, not completing surgical resection during the initial course of treatment, lower body mass index, diabetes, current smoking, and more pack-years smoked (Table S1).

**Table 1:** Characteristics of participants with incident colorectal cancer enrolled in the Southern Community Cohort Study (2002-2009)^a^.

Variables	Full sample (N = 1038)	Annual household income	
		< $15,000 (N = 609)	≥ $15,000 (N = 429)
Age at CRC diagnosis (years)	61 (56-68)	61 (56-69)	61 (56-68)
Enrollment source			
Health Centers	917 (88%)	590 (97%)	327 (76%)
Phone/mail	121 (12%)	19 (3%)	102 (24%)
Sex			
Female	597 (58%)	374 (61%)	223 (52%)
Male	441 (42%)	235 (39%)	206 (48%)
Race			
Black	756 (73%)	454 (75%)	302 (70%)
White	255 (25%)	138 (23%)	117 (27%)
Other^b^	27 (3%)	17 (3%)	10 (2%)
Education			
< HS graduate	353 (34%)	279 (46%)	74 (17%)
HS graduate	353 (34%)	206 (34%)	147 (34%)
Post-HS education	332 (32%)	124 (20%)	208 (48%)
Insurance coverage			
No	407 (39%)	275 (45%)	132 (31%)
Yes	631 (61%)	334 (55%)	297 (69%)
Marital status			
Married	342 (33%)	133 (22%)	209 (49%)
Separated or divorced	337 (32%)	228 (37%)	109 (25%)
Widowed	134 (13%)	95 (16%)	39 (9%)
Single, never married	225 (22%)	153 (25%)	72 (17%)
SEER stage			
Local	328 (32%)	175 (29%)	153 (36%)
Regional	277 (27%)	164 (27%)	113 (26%)
Distant	201 (19%)	120 (20%)	81 (19%)
Missing	232 (22%)	150 (25%)	82 (19%)
Tumor location			
Colon	683 (66%)	389 (64%)	294 (69%)
Rectum	220 (21%)	129 (21%)	91 (21%)
Missing	135 (13%)	91 (15%)	44 (10%)
CRC screening prior to diagnosis			
No	586 (56%)	361 (59%)	225 (52%)
Yes	452 (44%)	248 (41%)	204 (48%)
Surgical resection			
No	152 (15%)	102 (17%)	50 (12%)
Yes	695 (67%)	388 (64%)	307 (72%)
Missing	191 (18%)	119 (20%)	72 (17%)
Body mass index (kg/m2)			
<25.0	259 (25%)	170 (28%)	89 (21%)
25.0-29.9	316 (30%)	181 (30%)	135 (31%)
30.0-34.9	242 (23%)	124 (20%)	118 (28%)
35.0-39.9	115 (11%)	73 (12%)	42 (10%)
≥40.0	106 (10%)	61 (10%)	45 (10%)
Diabetes status			
No	773 (74%)	447 (73%)	326 (76%)
Yes	265 (26%)	162 (27%)	103 (24%)
Alcohol			
Non-drinker	540 (52%)	324 (53%)	216 (50%)
Light/mod. drinker^c^	326 (31%)	176 (29%)	150 (35%)
Heavy drinker^c^	172 (17%)	109 (18%)	63 (15%)
Smoking status			
Never	384 (37%)	217 (36%)	167 (39%)
Former	273 (26%)	137 (22%)	136 (32%)
Current	381 (37%)	255 (42%)	126 (29%)
Pack years smoked	6.6 (0.0-22.0)	7.4 (0.0-23.0)	5.0 (0.0-19.0)
Meeting physical activity guidelines			
No	853 (82%)	526 (86%)	327 (76%)
Yes^d^	185 (18%)	83 (14%)	102 (24%)
HEI score - 2010	58 (50-67)	57 (49-65)	61 (53-68)

Abbreviations: CRC – colorectal cancer; HEI – Healthy Eating Index; HS – high school; SEER – Surveillance, Epidemiology, and End Result.

a Data presented as median (interquartile range) for continuous variables, and as N (%) for categorical variables.

b ‘Other’ includes participants who self-reported Hispanic/Latino ethnicity or Asian/Pacific Islander, American Indian or Alaska Native racial identity, mixed racial ancestry, or ‘other’ racial identity.

c Light/moderate alcohol consumption defined as >0 but <1 drink/day for women and <2 drinks/day for men. Heavy alcohol consumption defined as at least ≥1 drink/day for women and ≥2 drinks/day for men.

d Defined as completing ≥150 min/week of moderate or vigorous activity.

Fifty-nine percent of participants reported having annual household incomes <$15,000/year (Table 1), while 8% had incomes ≥$50,000/year. Higher income at enrollment (≥$15,000/year) was associated with enrollment via phone/mail, male sex, higher education, and being married (Table 1, Table S2). A higher percentage of individuals with higher incomes (≥$15,000/year vs. < $15,000/year) reported having insurance coverage at enrollment (69% vs. 55%), completing CRC screening prior to diagnosis (48% vs. 41%), and undergoing surgical resection during the first course of treatment (72% vs. 64%). Higher income was not associated with tumor location but was associated with a higher proportion of localized tumor stage (36% vs. 29%) and a lower proportion of missing tumor stage (19% vs. 25%). Higher income was also related to higher adherence to guidelines for physical activity and diet, lower prevalence of current smoking, and fewer pack-years smoked (Table 1, Table S2).

Higher annual household income was dose-dependently associated with lower risk for overall and CRC-specific mortality (Table 2). After adjusting for potential mediating variables, including healthcare- and lifestyle-related mortality risk factors, higher income was still associated with lower risk for overall mortality (HR = 0.53, 95% CI: 0.34-0.83, ≥$50,000 vs. <$15,000). Higher income was no longer significantly associated with risk for CRC-specific mortality (HR = 0.69, 95%CI 0.40-1.20). To determine whether race modifies the impact of SES on mortality outcomes, the associations between income and mortality were stratified by self-identified racial identity. A protective association between income and overall mortality was identified for both Black-identifying (HR = 0.49, 95% CI: 0.27-0.88, ≥$50,000 vs. <$15,000) and White-identifying (HR = 0.20, 95% CI: 0.10-0.54, ≥$50,000 vs. <$15,000) participants, with similar associations observed for CRC-specific mortality (Table 2). The association between income and mortality was not modified by race, sex, or education level (all p-interaction>0.1).

**Table 2:** Hazard ratios (with 95% confidence intervals) for mortality by level of household income.

Income	Analytic Cohort (N = 1038)			Black Participants (N = 756)			White Participants (N = 255)		
	Events/total	HR (95% CI)^a^	HR (95% CI)^b^	Events/total	HR (95% CI)^a^	HR (95% CI)^b^	Events/total	HR (95% CI)^a^	HR (95% CI)^b^
Overall mortality									
< $15,000	387/609	1 (ref)	1 (ref)	290/454	1 (ref)	1 (ref)	87/138	1 (ref)	1 (ref)
$15,000-24,999	113/203	0.78 (0.63-0.97)	0.89 (0.71-1.11)	87/161	0.78 (0.61-1.01)	0.92 (0.71-1.19)	25/40	0.66 (0.41-1.06)	0.86 (0.50-1.46)
$25,000-49,999	76/148	0.68 (0.53-0.88)	0.68 (0.51-0.90)	61/106	0.79 (0.59-1.05)	0.78 (0.57-1.07)	14/36	0.34 (0.18-0.64)	0.37 (0.18-0.75)
≥ $50,000	30/78	0.42 (0.27-0.64)	0.53 (0.34-0.83)	13/35	0.49 (0.27-0.88)	0.44 (0.24-0.80)	17/41	0.20 (0.10-0.41)	0.54 (0.22-1.30)
CRC-specific mortality									
< $15,000	224/609	1 (ref)	1 (ref)	172/454	1 (ref)	1 (ref)	47/138	1 (ref)	1 (ref)
$15,000-24,999	71/203	0.82 (0.62-1.08)	1.00 (0.75-1.33)	57/161	0.85 (0.62-1.16)	1.08 (0.77-1.50)	13/40	0.58 (0.30-1.13)	0.72 (0.33-1.58)
$25,000-49,999	47/148	0.72 (0.52-1.00)	0.68 (0.47-0.98)	37/106	0.80 (0.55-1.15)	0.77 (0.51-1.17)	10/36	0.39 (0.18-0.85)	0.67 (0.26-1.69)
≥ $50,000	22/78	0.53 (0.32-0.88)	0.69 (0.40-1.20)	9/35	0.59 (0.29-1.20)	0.58 (0.27-1.26)	13/41	0.20 (0.08-0.52)	0.65 (0.20-2.15)

Abbreviations: CI – confidence interval; CRC – colorectal cancer; HR – hazard ratio.

a Adjusted for age (as time scale), sex, marital status, and enrollment source.

b Fully adjusted model – further adjusted for potential mediating variables including stage, surgery during the first course of treatment, insurance status, screening for colorectal cancer, diabetes duration, smoking history, alcohol consumption, Healthy Eating Index score, and meeting physical activity guidelines.

When household income was dichotomized at $15,000/year, income ≥$15,000 was associated with a HR of 0.70 (95%CI: 0.58-0.84) for overall mortality and 0.75 (95% CI: 0.59-0.94) for CRC-specific mortality compared to participants with income <$15,000 (Table 3). The association between income and mortality was mediated by differences in tumor stage and completion of surgical resection of the primary tumor site (overall mortality: 16%; CRC-specific mortality: 33%). Among Black-identifying participants with distant tumor stage, higher income (≥$15,000/year) was associated with higher completion of surgical resection compared to income < $15,000/year (71% vs. 52%, respectively), whereas the association between income and surgery among White-identifying participants with distant tumor stage was less pronounced. Participants with distant tumor stage were less likely to have completed surgical resection of the primary tumor site for both Black- and White-identifying participants (Table 4). Consequently, to mitigate the potential for mutual confounding, we investigate mediation by stage and surgery collectively.

**Table 3:** Hazard ratios for mortality by income level (≥$15,000 vs. <$15,000 (ref)), including adjustment for mediating variables.

Overall mortality	Full sample (N = 1038)		Local/regional tumors (N = 605)	
	HR (95%CI)	% change in logHR attributed to mediator	HR (95%CI)	% change in logHR attributed to mediator
Model 1 a (ref)	0.70 (0.58-0.84)	(Ref)	0.71 (0.54-0.94)	(Ref)
Stage and healthcare				
+stage and surgery	0.74 (0.62-0.89)	16%	0.73 (0.55-0.97)	7%
+insurance	0.69 (0.58-0.83)	−3%	0.70 (0.52-0.93)	−7%
+screening	0.68 (0.57-0.82)	−6%	0.69 (0.52-0.92)	−9%
*+all stage and healthcare*^b^	0.73 (0.61-0.88)	13%	0.70 (0.52-0.93)	−7%
Lifestyle				
+diabetes years	0.72 (0.60-0.86)	7%	0.74 (0.56-0.99)	13%
+smoking	0.73 (0.61-0.88)	11%	0.75 (0.56-1.00)	15%
+alcohol	0.71 (0.59-0.85)	2%	0.71 (0.54-0.94)	0%
+Healthy Eating Index score	0.72 (0.60-0.87)	9%	0.75 (0.57-1.00)	16%
+physical activity	0.70 (0.58-0.84)	−2%	0.70 (0.52-0.93)	−7%
*+all mediators*^c^	0.77 (0.63-0.93)	26%	0.78 (0.57-1.06)	27%
**CRC-specific mortality**				
Model 1 a (ref)	0.75 (0.59-0.94)	(Ref)	0.75 (0.49-1.16)	(Ref)
Stage and healthcare				
+stage and surgery	0.82 (0.65-1.05)	33%	0.82 (0.53-1.28)	31%
+insurance	0.75 (0.59-0.95)	−1%	0.73 (0.47-1.14)	−9%
+screening	0.74 (0.58-0.93)	−5%	0.74 (0.48-1.15)	−5%
*+all stage and healthcare*^b^	0.83 (0.65-1.06)	36%	0.80 (0.51-1.25)	20%
Lifestyle				
+diabetes years	0.76 (0.60-0.96)	5%	0.76 (0.49-1.18)	6%
+smoking	0.77 (0.61-0.97)	9%	0.80 (0.51-1.24)	20%
+alcohol	0.76 (0.60-0.95)	3%	0.76 (0.49-1.17)	3%
+Healthy Eating Index score	0.77 (0.61-0.97)	9%	0.79 (0.51-1.23)	19%
+physical activity	0.74 (0.59-0.94)	−3%	0.76 (0.49-1.18)	4%
*+all mediators*^c^	0.85 (0.66-1.09)	44%	0.90 (0.55-1.45)	62%

Abbreviations: CRC – colorectal cancer; HR – hazard ratio; CI – confidence interval.

a Each mediator is individually to Model 1 which is adjusted for age (as time-scale), sex, race, enrollment source, and marital status.

b Adjusted for stage, surgical resection of the primary tumor site, insurance status, and screening.

c Adjusted for stage, surgical resection of the primary tumor site, insurance status, and screening, diabetes duration, smoking history, alcohol consumption, Healthy Eating Index score, and physical activity.

**Table 4:** Cross-tabulation of tumor stage and surgical resection of the primary tumor, by race and income.

Black, low income^a^ (<$15,000/year, N = 454)	SEER stage			
	Local	Regional	Distant	Missing
*Surgery*^a^	N = 131	N = 121	N = 92	N = 110
No (N = 79)	11 (2%)	9 (2%)	42 (9%)	17 (4%)
Yes (N = 295)	117 (26%)	111 (24%)	48 (11%)	19 (4%)
Missing (N = 80)	3 (1%)	1 (0%)	2 (0%)	74 (16%)
**Black, high income^a^ (≥ $15,000/year, N=302)**				
*Surgery*^a^	N = 107	N = 79	N = 58	N = 58
No (N = 39)	8 (3%)	3 (1%)	16 (5%)	12 (4%)
Yes (N = 223)	94 (31%)	76 (25%)	41 (14%)	12 (4%)
Missing (N = 40)	5 (2%)	0 (0%)	1 (0%)	34 (11%)
**White, low income^a^ (< $15,000/year, N=138)**				
*Surgery*^a^	N = 40	N = 36	N = 24	N = 38
No (N = 18)	4 (3%)	1 (1%)	10 (7%)	3 (2%)
Yes (N = 82)	31 (22%)	32 (23%)	14 (10%)	5 (4%)
Missing (N = 38)	5 (4%)	3 (2%)	0 (0%)	30 (22%)
**White, high income^a^ (≥ $15,000/year, N=117)**				
*Surgery*^a^	N = 43	N = 32	N = 22	N = 20
No (N = 11)	3 (3%)	0 (0%)	7 (6%)	1 (1%)
Yes (N = 76)	33 (28%)	27 (23%)	14 (12%)	2 (2%)
Missing (N = 30)	7 (6%)	5 (4%)	1 (1%)	17 (15%)

Abbreviations: SEER – Surveillance, Epidemiology, and End Results.

a Refers to surgical resection in the first course of treatment.

**Fig. 1:**
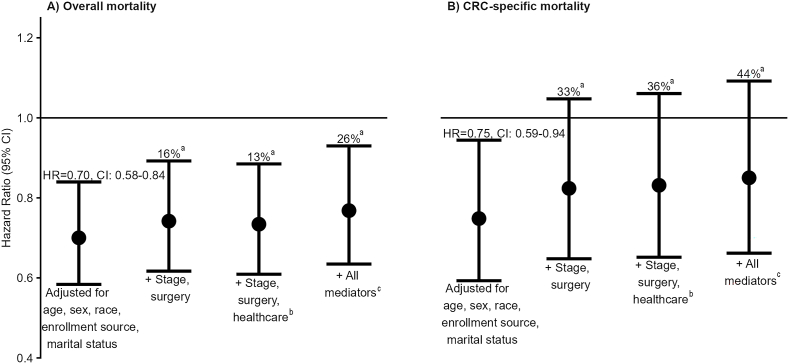
Hazard ratios for mortality by income level (≥$15,000 vs. &lt;$15,000 (ref)), including adjustment for mediating variables. Abbreviations: CI – confidence interval; CRC – colorectal cancer; HEI – Healthy Eating Index ^a^ Percentages reflect the indirect effect of the mediating variables, i.e. the proportion of the log hazard ratio for income that is explained by adjustment for the mediating variables. ^b^Further adjusted for healthcare-related mediators include screening for colorectal cancer, insurance status at enrollment, and surgical resection of the primary tumor. ^c^ Further adjusted for healthcare-related mediators (see b) and for lifestyle-related mediators including duration of diabetes, smoking status, alcohol consumption, Healthy Eating Index score, and physical activity.

Smoking history also mediated the associations between income and mortality, weakening the association with overall mortality by 11% (HR = 0.73, 95% CI: 0.61-0.88) and 9% for CRC-specific mortality (HR = 0.77, 95%CI: 0.61-0.97). Differences in the Healthy Eating Index score also explained 9% of the associations between income and overall, CRC-specific mortality. Other individual variables contributed minimally to the mediation of income with mortality outcomes. The cumulative mediation influence of all variables examined explained 26% of the association between income and overall mortality and 44% with CRC-specific mortality (Table 3, Fig. 1).

After excluding participants with distant tumors at diagnosis or missing tumor stage, associations between income (≥$15,000 vs < $15,000) and mortality were similar compared to the full sample (overall mortality: HR = 0.71, 95% CI: 0.54-0.94; CRC-specific mortality: HR = 0.75, 95% CI: 0.49-1.16, Table 3). For overall mortality, the association with income was explained in part by differences in diabetes status (13%), smoking history (15%), and Healthy Eating Index score (16%). For CRC-specific mortality, the association was mediated by differences in stage and surgery (31%), smoking status (20%), and the Healthy Eating Index score (19%). Adjustment for all potential mediators explained 27% and 62% of the associations with overall, CRC-specific mortality, respectively (Table 3, Fig. S1).

## 5 Discussion

In this racially and SES diverse cohort of individuals with incident CRC, household income is inversely associated with overall and CRC-specific mortality. Nationally, socioeconomic factors contribute to racial and geographic disparities for CRC (Carethers & Doubeni, 2020). The poverty rate is higher in the US Black population (17.8%) compared to the White population (9.7%) (United States Census Bureau, n.d.) contributing to the 29% higher rate of CRC mortality in the US Black population (Siegel et al., 2026). High-poverty areas, such as the Lower Mississippi Delta (Siegel et al., 2015) have elevated CRC incidence and mortality rates as do rural compared to urban areas (Zahnd et al., 2021). The current study adds to this data by quantifying the impact of socioeconomic disparities on CRC outcomes and identifying underlying causes. We find the protective association for higher SES is largely explained by differences in the early detection of CRC and the completion of surgical resection of the primary tumor site.

This study is informed by and consistent with Fundamental Causes Theory, which seeks to understand how socioeconomic disparities in disease arise due to differences in knowledge and access to resources, such as cancer screening and treatment (Link & Phelan, 1995). Fundamental Causes Theory provides a valuable framework for identifying root causes of health disparities, and therefore for designing and implementing policies and population-level interventions to promote health equity (Link & Phelan, 1995). This study also fits into the emerging PCIBM framework for cancer research, an approach that seeks to utilize tumor biobanks in longitudinal studies to understand how cancer risk factors influence the molecular characteristics of cancer (Ogino et al., 2026). In this framework, diagnosis stage is a pathobiological characteristic that reflects aggressive molecular characteristics that impact cancer progression and mortality (Coebergh van den Braak et al., 2020). The SCCS is a valuable resource for investigating how socioeconomic and lifestyle characteristics impact the distribution of aggressive tumor molecular characteristics in CRC, and this is a promising avenue for additional research.

Our finding of an inverse association between household income and CRC mortality is consistent with results from the National Cancer Database, where participants with higher incomes (≥30,000/year) had lower risk for overall mortality compared to individuals with incomes less than $30,000 (6-40% lower risk), with consistent results across age groups (Gabriel et al., 2018). Together, these results indicate that modest improvements in SES for individuals with the lowest incomes could substantially impact CRC mortality.

In addition to the impact of income on mortality, we find stage at diagnosis and surgical resection of the primary tumor site are collectively the most important mediators of the association between income and mortality, explaining 33% of the association with CRC-specific mortality. This finding is consistent with Fundamental Causes Theory, as individuals with higher SES are expected to access healthcare resources at higher rates (Gulati et al., 2025), leading to early cancer detection and favorable treatment outcomes. As anticipated, we find that higher income is associated with lower odds of distant tumor stage and a higher likelihood of completing surgery for the present sample. Further, we identified an association between diagnosis stage and surgery that was influenced by SES, specifically among Black-identifying participants. An association between higher SES and more aggressive treatment of metastatic disease is consistent with previously published studies of metastatic CRC (Ahmed et al., 2022; Sell et al., 2020) and may contribute to better survival outcomes for higher-SES individuals. Considered together, results from the present analysis suggest that even modest SES-related differences in early cancer detection and treatment may contribute substantially to disparities in CRC mortality.

Results from other large, nationally representative studies support associations between higher SES, less invasive stage of CRC, and surgical treatment. In the National Cancer Database, higher SES encompassing income and education was associated with a lower prevalence of stage IV disease among adults age 18-40 years at CRC diagnosis (27.7% vs. 32.8% for high vs. low SES, respectively) (Salem et al., 2021). Likewise, results from the SEER database demonstrate that individuals residing in neighborhoods with higher median household income were less likely to have stage IV disease compared to individuals from low SES neighborhoods (Zhu et al., 2023). Further, other studies demonstrate that higher income is related to the completion of surgical resection (Alty et al., 2021; Fields et al., 2020). In the National Cancer Database, higher income is associated with lower refusal of surgery among eligible patients with stage III colon cancer (OR = 0.39, 95% CI: 0.20-0.74, income ≥$63,333/year vs. <$40,227) (Alty et al., 2021), with similar results for rectal cancer (Fields et al., 2020). Additionally, individuals with higher incomes are more likely to receive resection of colorectal cancer liver metastases (OR = 1.20, 95% CI: 1.07-1.37, income quartile four vs. one), indicating more aggressive surgical intervention among higher SES populations. Other research from the National Cancer Database demonstrates that higher income is associated with greater likelihood of receiving guideline concordant care for stage II-III colon cancer (Honaker et al., 2023). Together, these findings support earlier stage and optimal treatment of CRC as important for outcomes and contributors to SES disparities in CRC. The same studies demonstrate that Black racial identity is independently associated with advanced diagnosis stage and lower odds of surgical resection (Ahmed et al., 2022; Alty et al., 2021; Fields et al., 2020; Salem et al., 2021), but have not reported whether race modifies the impact of SES on these outcomes. Among participants with distant tumor stage, we find that higher income is related to the completion of surgical resection among Black-identifying participants specifically, suggesting more aggressive treatment. This finding requires corroboration in an independent cohort.

CRC screening is facilitated by insurance coverage and linked to lower risk of CRC incidence, mortality and late stage at diagnosis (Gupta, 2022). Fundamental Causes Theory highlights how high-SES individuals can access insurance coverage and healthcare at higher rates (Gulati et al., 2025), leading to higher rates of preventive screening (Clouston et al., 2020). Consistent with this underlying theory, individuals with lower SES are less likely to complete CRC screening (Luo et al., 2023) contributing to the higher likelihood of distant tumor stage at diagnosis. Likewise, we find that SCCS participants with lower incomes are less likely to report having insurance coverage at enrollment or completing endoscopy or stool-based testing. Notably, the difference in screening prevalence is modest and neither insurance coverage nor screening mediate the associations between income and mortality. Changes in health policy may have increased insurance coverage rates as well as the availability of preventive and diagnostic screening among low-income SCCS participants after study enrollment. For example, the Affordable Care Act (ACA) expanded coverage and removed cost-sharing by Medicare and private insurance for preventive screening in 2011 (Richman et al., 2016). Emerging evidence indicates that screening participation increased after passage of the ACA, especially among low-SES individuals (Richman et al., 2016a). Further, Medicaid expansion took effect in 2014 and afterwards for six of the SCCS recruitment states (KFF, 2025), and epidemiological evidence indicates that states that accepted Medicaid expansion had greater increases in endoscopy screening rates compared to states that declined (Lyu & Wehby, 2019). It is estimated that maximizing adherence to screening guidelines could prevent 68% of all CRC-related deaths in the US via removal of precancerous polyps and early detection of malignant tumors (Sharma et al., 2020). While screening does not mediate the association between income and mortality in this sample that developed incident CRC, increasing screening is essential to reduce the population burden of CRC and address socioeconomic disparities (Carethers & Doubeni, 2020).

Despite seeing differences in the rate of insurance coverage in high-versus low-income groups, we do not find that insurance mediates the association between income and CRC mortality in the present sample. This may reflect expanding coverage due to the ACA during the SCCS follow-up period (Richman et al., 2016), variability in insurance coverage, as well as the role of insurance copays, can create a barrier to timely treatment among individuals with insurance but fewer economic resources (Shankaran & Ramsey, 2015). Likewise, other barriers to treatment that are independent of insurance coverage may also contribute to treatment delays and worse outcomes for individuals with low SES. For example, these include financial and economic barriers such as loss of income from work, indirect costs accrued during treatment (e.g. transportation and hotel stay), and caregiving costs (Jafari et al., 2024). Other barriers to timely treatment may include local provider shortages, lack of transportation, fragmented care systems, and the administrative burden associated with hospital scheduling and paperwork (Bourgeois et al., 2024). Information about these barriers is not currently available from the SCCS.

Fundamental Causes Theory also posits that individuals with greater financial or knowledge resources will more readily adapt guidelines for healthy lifestyle behaviors, including not smoking, limiting alcohol consumption, and following a healthy diet pattern (Phelan et al., 2010). These lifestyle behaviors may improve prognosis or reduce risk for comorbidities that are linked to mortality (e.g. type 2 diabetes). Consistent with this, we find that individuals with higher SES report better adherence to guidelines for diet and physical activity, lower levels of current smoking and fewer pack years smoked, and a lower prevalence of self-reported diabetes. Further, differences in healthy lifestyle behaviors and comorbidities may partially mediate the association between higher income and lower risk for mortality among individuals with CRC, especially for those with local or regional tumor stage. We find that differences in smoking history (smoking status and pack years) mediated approximately 10% of the associations between income and mortality. Meta-analyses have demonstrated that pre-diagnostic smoking is associated with approximately 15-40% increased overall and CRC-specific mortality compared to non-smoking and that cessation mitigates risk (Botteri et al., 2008; Liang et al., 2009; Ordóñez-Mena et al., 2018; Walter et al., 2015). Higher smoking prevalence is also related to lower socioeconomic status (Zhang et al., 2021) and therefore public health interventions targeting smoking cessation may reduce the population burden of CRC mortality and mitigate disparities. Epidemiological research also supports an association between higher alcohol consumption and greater risk for CRC mortality among individuals consuming ≥30 g/day (approximately two drinks) (Cai et al., 2014), as well as protective associations for following a healthy diet pattern (e.g. the Healthy Eating Index or the Mediterranean Diet) (Castro-Espin & Agudo, 2022) and performing physical activity (Choy et al., 2022). Type 2 diabetes has been linked to higher risk for CRC mortality (Ling et al., 2021), including in the SCCS where self-reported type 2 diabetes was associated with 36% (CI: 6-74%) increased risk for CRC-specific mortality (Lawler et al., 2024). In the full sample, we find limited mediation of the income-mortality associations by health behaviors and comorbidities, although differences in the Healthy Eating Index score explained 9% of the association with both overall and CRC-specific mortality. There was stronger evidence for mediation by lifestyle-related factors among participants with local or regional tumors only (Table 3, Fig. S1). In this subgroup, smoking history and diet explained 20% and 19% of the association between income and CRC-specific mortality, respectively, while diabetes mediated 13% of the association with overall mortality. These findings indicate that the impact of lifestyle on mortality may be more pronounced in non-metastatic disease where survival rates are higher.

Our analysis includes several limitations. First, data about surgical resection is limited to the first course of treatment only. It is possible that participants received additional treatments which we are unaware of. Further, most SCCS participants have lower SES, making it difficult to compare mortality risk for individuals with high versus low SES. Consequently, the results may not generalize to populations with greater socioeconomic variability. However, the unique SCCS study design makes it possible to investigate how modest differences in income and associated lifestyle- and healthcare-related factors influence mortality. It is also possible that measurement error for lifestyle-related exposures (e.g. smoking history, diet, physical activity, diabetes, and alcohol consumption) caused us to underestimate their impact on the association between income and mortality. Strengths include the utilization of a sample enriched for non-Hispanic Black participants, who are underrepresented in studies of CRC despite elevated mortality risk.

## 6 Conclusions

In the current manuscript we quantify how stage, healthcare- and lifestyle-related risk factors contribute to socioeconomic disparities for CRC mortality and find that social determinants related to access and use of healthcare to be of the highest importance. Our findings indicate that interventions to promote equitable access to CRC screening and curative surgery will mitigate SES-related disparities for CRC mortality, especially among the Black population.

## Ethical statement

All participants provided written informed consent. The Institutional Review Boards of the Vanderbilt University Medical Center and Meharry Medical College provided approval for this study. All study activities conformed to the tenets of the Declaration of Helsinki.

## Funding

This work was supported by the National Cancer Institute at the National Institutes of Health (grant number R01 CA255318 to S Warren Andersen); the University of Wisconsin-Madison, Office of Vice Chancellor for Research and Graduate Education with funding from the Wisconsin Alumni Research Foundation and the University of Wisconsin Carbone Cancer (grant number P30 CA014520 to HH Bailey). The Southern Community Cohort Study (SCCS) is supported by the National Cancer Institute at the National Institutes of Health (grant number U01 CA202979 to W Zheng), including special allocations from the American Recovery and Reinvestment Act (grant number 3R01 CA092447-08S1 to WJ Blot). The funders had no role in study design, data collection and analysis, decision to publish, or preparation of the manuscript.

## CRediT authorship contribution statement

**Thomas P. Lawler:** Formal analysis, Investigation, Methodology, Visualization, Writing – original draft. **Oluwatoyosi Ogunmuyiwa:** Writing – review & editing. **Rida A. Khatri:** Writing – review & editing. **Mark D. Steinwandel:** Data curation, Writing – review & editing. **Ronald E. Gangnon:** Methodology, Writing – review & editing. **Martha J. Shrubsole:** Writing – review & editing. **Shaneda Warren Andersen:** Conceptualization, Funding acquisition, Methodology, Project administration, Resources, Supervision, Writing – review & editing.

## Declaration of competing interest

The authors declare that they have no known competing financial interests or personal relationships that could have appeared to influence the work reported in this paper.

## Data Availability

Data were analyzed under license from the SCCS. Data are available to qualified researchers via request through the SCCS website (https://www.southerncommunitystudy.org/).
