# Supplementary material for: Identifying clinical and lifestyle factor mediators of the association between socioeconomic status and colorectal cancer mortality
Source: SSM Popul Health. 2026 Jul 18;35:101952. doi: 10.1016/j.ssmph.2026.101952 (PMC13393643; doi:10.1016/j.ssmph.2026.101952)
Supplement: Multimedia component 1 [file mmc1.docx]

| **Table S1:** Characteristics of participants with incident colorectal cancer enrolled in the Southern Community Cohort Study between 2002-2009, stratified by mortality status at the end of follow-up^a^ | | | | | | | |  |
| --- | --- | --- | --- | --- | --- | --- | --- | --- |
|  | **Overall mortality** | | | |  | **CRC-specific mortality** | |  |
| **Variables** | **Alive**  **(N = 432)** | | **Deceased**  **(N = 606)** | |  | **Yes**  **(N = 364)** | |  |
| Age at CRC diagnosis (years) |  | |  | |  |  | |  |
| 40-49 | 26 (39%) | | 40 (61%) | |  | 26 (39%) | |  |
| 50-54 | 72 (49%) | | 76 (51%) | |  | 41 (28%) | |  |
| 55-59 | 112 (47%) | | 128 (53%) | |  | 82 (34%) | |  |
| 60-64 | 87 (48%) | | 94 (52%) | |  | 55 (30%) | |  |
| 65-69 | 74 (40%) | | 111 (60%) | |  | 68 (37%) | |  |
| ≥ 70 | 61 (28%) | | 157 (72%) | |  | 92 (42%) | |  |
| Enrollment source |  | |  | |  |  | |  |
| Community Health Centers | 372 (41%) | | 545 (59%) | |  | 322 (35%) | |  |
| Phone/mail | 60 (50%) | | 61 (50%) | |  | 42 (35%) | |  |
| Sex |  | |  | |  |  | |  |
| Female | 270 (45%) | | 327 (55%) | |  | 196 (33%) | |  |
| Male | 162 (37%) | | 279 (63%) | |  | 168 (38%) | |  |
| Race |  | |  | |  |  | |  |
| Black/African American | 305 (40%) | | 451 (60%) | |  | 275 (36%) | |  |
| White | 112 (44%) | | 143 (56%) | |  | 83 (33%) | |  |
| Other^b^ | 15 (56%) | | 12 (44%) | |  | 6 (22%) | |  |
| Education |  | |  | |  |  | |  |
| < HS graduate | 130 (37%) | | 223 (63%) | |  | 121 (34%) | |  |
| HS graduate | 148 (42%) | | 205 (58%) | |  | 126 (36%) | |  |
| Post-HS education | 154 (46%) | | 178 (54%) | |  | 117 (35%) | |  |
| Insurance coverage |  | |  | |  |  | |  |
| No | 177 (43%) | | 230 (57%) | |  | 146 (36%) | |  |
| Yes | 255 (40%) | | 376 (60%) | |  | 218 (35%) | |  |
| Marital status |  | |  | |  |  | |  |
| Married | 169 (49%) | | 173 (51%) | |  | 106 (31%) | |  |
| Separated or divorced | 135 (40%) | | 202 (60%) | |  | 129 (38%) | |  |
| Widowed | 41 (31%) | | 93 (69%) | |  | 45 (34%) | |  |
| Single, never married | 87 (39%) | | 138 (61%) | |  | 84 (37%) | |  |
| Stage |  | |  | |  |  | |  |
| Local | 207 (63%) | | 121 (37%) | |  | 36 (11%) | |  |
| Regional | 143 (52%) | | 134 (48%) | |  | 69 (25%) | |  |
| Distant | 15 (7%) | | 186 (93%) | |  | 148 (74%) | |  |
| Missing | 67 (29%) | | 165 (71%) | |  | 111 (48%) | |  |
| Tumor location |  | |  | |  |  | |  |
| Colon | 308 (45%) | | 375 (55%) | |  | 219 (32%) | |  |
| Rectum | 99 (45%) | | 121 (55%) | |  | 71 (32%) | |  |
| Missing | 25 (19%) | | 110 (81%) | |  | 74 (55%) | |  |
| CRC screening prior to diagnosis |  | |  | |  |  | |  |
| No | 237 (40%) | | 349 (60%) | |  | 219 (37%) | |  |
| Yes | 195 (43%) | | 257 (57%) | |  | 145 (32%) | |  |
| Surgical resection |  | |  | |  |  | |  |
| No | 21 (14%) | | 131 (86%) | |  | 90 (59%) | |  |
| Yes | 364 (52%) | | 331 (48%) | |  | 178 (26%) | |  |
| Missing | 47 (25%) | | 144 (75%) | |  | 96 (50%) | |  |
| Body mass index (kg/m2) |  | |  | |  |  | |  |
| < 25.0 | 99 (38%) | | 160 (62%) | |  | 96 (37%) | |  |
| 25.0-29.9 | 123 (39%) | | 193 (61%) | |  | 121 (38%) | |  |
| 30.0-34.9 | 102 (42%) | | 140 (58%) | |  | 86 (36%) | |  |
| 35.0-39.9 | 54 (47%) | | 61 (53%) | |  | 35 (30%) | |  |
| ≥ 40.0 | 54 (51%) | | 52 (49%) | |  | 26 (25%) | |  |
| Diabetes status |  | |  | |  |  | |  |
| No | 343 (44%) | | 430 (56%) | |  | 269 (35%) | |  |
| Yes | 89 (34%) | | 176 (66%) | |  | 95 (36%) | |  |
| Alcohol |  | |  | |  |  | |  |
| Non-drinker | 221 (41%) | | 319 (59%) | |  | 186 (34%) | |  |
| Light/moderate drinker^c^ | 152 (47%) | | 174 (53%) | |  | 108 (33%) | |  |
| Heavy drinker^c^ | 59 (34%) | | 113 (66%) | |  | 70 (41%) | |  |
| Smoking status |  | |  | |  |  | |  |
| Never | 177 (46%) | | 207 (54%) | |  | 133 (35%) | |  |
| Former | 114 (42%) | | 159 (58%) | |  | 97 (36%) | |  |
| Current | 141 (37%) | | 240 (63%) | |  | 134 (35%) | |  |
| Pack years smoked |  | |  | |  |  | |  |
| 0 (non-smoker) | 175 (46%) | | 205 (54%) | |  | 131 (34%) | |  |
| 0.03 – 7.5 | 70 (42%) | | 98 (58%) | |  | 63 (38%) | |  |
| 7.6 – 16.5 | 75 (46%) | | 87 (54%) | |  | 51 (31%) | |  |
| 17.0 – 32.0 | 61 (36%) | | 110 (64%) | |  | 58 (34%) | |  |
| 32.3 – 170.0 | 51 (32%) | | 106 (68%) | |  | 61 (39%) | |  |
| Meeting physical activity guidelines |  | |  | |  |  | |  |
| No | 352 (41%) | | 501 (59%) | |  | 297 (35%) | |  |
| Yes^d^ | 80 (43%) | | 105 (57%) | |  | 67 (36%) | |  |
| HEI score – 2010 (quintiles) |  | |  | |  |  | |  |
| Q1 (22.5 – 47.9) | 85 (41%) | | 122 (59%) | |  | 69 (33%) | |  |
| Q2 (47.9 – 55.8) | 84 (40%) | | 124 (60%) | |  | 76 (37%) | |  |
| Q3 (55.8 – 61.3) | 85 (38%) | | 137 (62%) | |  | 83 (37%) | |  |
| Q4 (61.3 – 68.8) | 80 (41%) | | 114 (59%) | |  | 72 (37%) | |  |
| Q5 (68.9 – 93.4) | 98 (47%) | | 109 (53%) | |  | 64 (31%) | |  |
| Abbreviations: CRC – colorectal cancer; Healthy Eating Index; HS – high school  ^a^ Data presented as median (interquartile range) for continuous variables, and as N (%) for categorical variables  ^b^ Includes Hispanic/Latino, Asian or Pacific Islander, American Indian or Alaska Native, Other racial or ethnic group, or 2+ race  ^c^ Light/moderate alcohol consumption defined as greater >0 but <1 drink/day for women and <2 drinks/day for men. Heavy alcohol consumption defined as ≥1 drink/day for women and ≥2 drinks/day for men.  ^d^ Defined as completing ≥150 minutes/week of moderate or vigorous activity. | | | | | | | |  |
| **Table S2:** Odds ratios (with 95% confidence intervals) for higher annual household income (≥ $15,000/year) by level of demographic, clinical, and lifestyle risk factors for mortality (N = 1,038) | | | | | | | | |
|  | | **Annual household income** | | | | | | |
| **Variables** | | < $15,000  (N = 609) | | ≥ $15,000  (N = 429) | | | OR  (95% CI)^a^ | |
| Age at CRC diagnosis (years) | |  | |  | | |  | |
| 40-49 | | 33 (50%) | | 33 (50%) | | | 1 (ref) | |
| 50-54 | | 94 (64%) | | 54 (36%) | | | 0.49 (0.26-0.93) | |
| 55-59 | | 138 (58%) | | 102 (43%) | | | 0.67 (0.37-1.20) | |
| 60-64 | | 107 (59%) | | 74 (41%) | | | 0.52 (0.28-0.96) | |
| 65-69 | | 98 (53%) | | 87 (47%) | | | 0.64 (0.35-1.18) | |
| ≥ 70 | | 139 (64%) | | 79 (36%) | | | 0.39 (0.21-0.73) | |
| Enrollment source | |  | |  | | |  | |
| Health Centers | | 590 (64%) | | 327 (36%) | | | 1 (ref) | |
| Phone/mail | | 19 (16%) | | 102 (84%) | | | 8.40 (4.92-14.34) | |
| Sex | |  | |  | | |  | |
| Female | | 374 (63%) | | 223 (37%) | | | 1 (ref) | |
| Male | | 235 (53%) | | 206 (47%) | | | 1.13 (0.85-1.50) | |
| Race | |  | |  | | |  | |
| Black/African American | | 454 (60%) | | 302 (40%) | | | 1 (ref) | |
| White | | 138 (54%) | | 117 (46%) | | | 0.91 (0.65-1.26) | |
| Other^b^ | | 17 (63%) | | 10 (37%) | | | 0.65 (0.26-1.59) | |
| Education | |  | |  | | |  | |
| < HS graduate | | 279 (79%) | | 74 (21%) | | | 1 (ref) | |
| HS graduate | | 206 (58%) | | 147 (42%) | | | 2.90 (2.01-4.17) | |
| Post-HS education | | 124 (37%) | | 208 (63%) | | | 6.51 (4.44-9.55) | |
| Insurance coverage | |  | |  | | |  | |
| No | | 275 (68%) | | 132 (32%) | | | 1 (ref) | |
| Yes | | 334 (53%) | | 297 (47%) | | | 1.64 (1.22-2.20) | |
| Marital status | |  | |  | | |  | |
| Married | | 133 (39%) | | 209 (61%) | | | 1 (ref) | |
| Separated or divorced | | 228 (68%) | | 109 (32%) | | | 0.34 (0.24-0.47) | |
| Widowed | | 95 (71%) | | 39 (29%) | | | 0.36 (0.23-0.58) | |
| Single, never married | | 153 (68%) | | 72 (32%) | | | 0.35 (0.24-0.51) | |
| Stage | |  | |  | | |  | |
| Local | | 175 (53%) | | 153 (47%) | | | 1 (ref) | |
| Regional | | 164 (59%) | | 113 (41%) | | | 0.81 (0.57-1.15) | |
| Distant | | 120 (60%) | | 81 (40%) | | | 0.76 (0.51-1.12) | |
| Missing | | 150 (65%) | | 82 (35%) | | | 0.68 (0.46-0.99) | |
| Tumor location | |  | |  | | |  | |
| Colon | | 389 (57%) | | 294 (43%) | | | 1 (ref) | |
| Rectum | | 129 (59%) | | 91 (41%) | | | 0.85 (0.61-1.20) | |
| Missing | | 91 (67%) | | 44 (33%) | | | 0.74 (0.48-1.12) | |
| CRC screening prior to diagnosis | |  | |  | | |  | |
| No | | 361 (62%) | | 225 (38%) | | | 1 (ref) | |
| Yes | | 248 (55%) | | 204 (45%) | | | 1.21 (0.91-1.61) | |
| Surgical resection | |  | |  | | |  | |
| No | | 102 (67%) | | 50 (33%) | | | 1 (ref) | |
| Yes | | 388 (56%) | | 307 (44%) | | | 1.35 (0.91-2.02) | |
| Missing | | 119 (62%) | | 72 (38%) | | | 1.16 (0.71-1.89) | |
| Body mass index (kg/m2) | |  | |  | | |  | |
| < 25.0 | | 170 (66%) | | 89 (34%) | | | 1 (ref) | |
| 25.0-29.9 | | 181 (57%) | | 135 (43%) | | | 1.35 (0.93-1.97) | |
| 30.0-34.9 | | 124 (51%) | | 118 (49%) | | | 1.90 (1.27-2.84) | |
| 35.0-39.9 | | 73 (63%) | | 42 (37%) | | | 1.10 (0.66-1.84) | |
| ≥ 40.0 | | 61 (58%) | | 45 (42%) | | | 1.56 (0.93-2.61) | |
| Diabetes status | |  | |  | | |  | |
| No | | 447 (58%) | | 326 (42%) | | | 1 (ref) | |
| Yes | | 162 (61%) | | 103 (39%) | | | 0.95 (0.69-1.30) | |
| Alcohol | |  | |  | | |  | |
| Non-drinker | | 324 (60%) | | 216 (40%) | | | 1 (ref) | |
| Light/moderate drinker^c^ | | 176 (54%) | | 150 (46%) | | | 1.20 (0.88-1.64) | |
| Heavy drinker^c^ | | 109 (63%) | | 63 (37%) | | | 0.80 (0.53-1.20) | |
| Smoking status | |  | |  | | |  | |
| Never | | 217 (57%) | | 167 (43%) | | | 1 (ref) | |
| Former | | 137 (50%) | | 136 (50%) | | | 1.19 (0.84-1.69) | |
| Current | | 255 (67%) | | 126 (33%) | | | 0.63 (0.45-0.87) | |
| Pack years smoked | |  | |  | | |  | |
| 0 (non-smoker) | | 214 (56%) | | 166 (44%) | | | 1 (ref) | |
| 0.03 – 7.5 | | 95 (57%) | | 73 (43%) | | | 0.98 (0.66-1.46) | |
| 7.6 – 16.5 | | 99 (61%) | | 63 (39%) | | | 0.79 (0.52-1.20) | |
| 17.0 – 32.0 | | 96 (56%) | | 75 (44%) | | | 0.94 (0.63-1.42) | |
| 32.3 – 170.0 | | 105 (67%) | | 52 (33%) | | | 0.58 (0.37-0.91) | |
| Meeting physical activity guidelines | |  | |  | | |  | |
| No | | 526 (62%) | | 327 (38%) | | | 1 (ref) | |
| Yes^d^ | | 83 (45%) | | 102 (55%) | | | 1.84 (1.29-2.62) | |
| HEI score – 2010 (quintiles) | |  | |  | | |  | |
| Q1 (22.5 – 47.9) | | 134 (65%) | | 73 (35%) | | | 1 (ref) | |
| Q2 (47.9 – 55.8) | | 131 (63%) | | 77 (37%) | | | 1.08 (0.69-1.68) | |
| Q3 (55.8 – 61.3) | | 133 (60%) | | 89 (40%) | | | 1.24 (0.80-1.91) | |
| Q4 (61.3 – 68.8) | | 107 (55%) | | 87 (45%) | | | 1.78 (1.14-2.78) | |
| Q5 (68.9 – 93.4) | | 104 (50%) | | 103 (50%) | | | 2.07 (1.32-3.23) | |
| Abbreviations: CI – confidence interval; CRC – colorectal cancer; HEI – Healthy Eating Index; HS – high school; OR – odds ratio  ^a^ Odds ratios are adjusted for age at diagnosis, race, sex, enrollment source, and marital status  ^b^ Includes Hispanic/Latino, Asian or Pacific Islander, American Indian or Alaska Native, Other racial or ethnic group, or 2+ race  ^c^ Light/moderate alcohol consumption defined as >0 but <1 drink/day for women and <2 drinks/day for men. Heavy alcohol consumption defined as ≥1 drink/day for women and ≥2 drinks/day for men.  ^d^ Defined as completing ≥150 minutes/week of moderate or vigorous activity. | | | | | | | | |

**Figure S1:** Hazard ratios (with 95% confidence intervals) for mortality outcomes for participants with annual household incomes ≥$15,000 compared to participants with annual incomes <$15,000, including cumulative adjustment for potential mediating variables, limited to participants with local or regional tumors only (N=589)


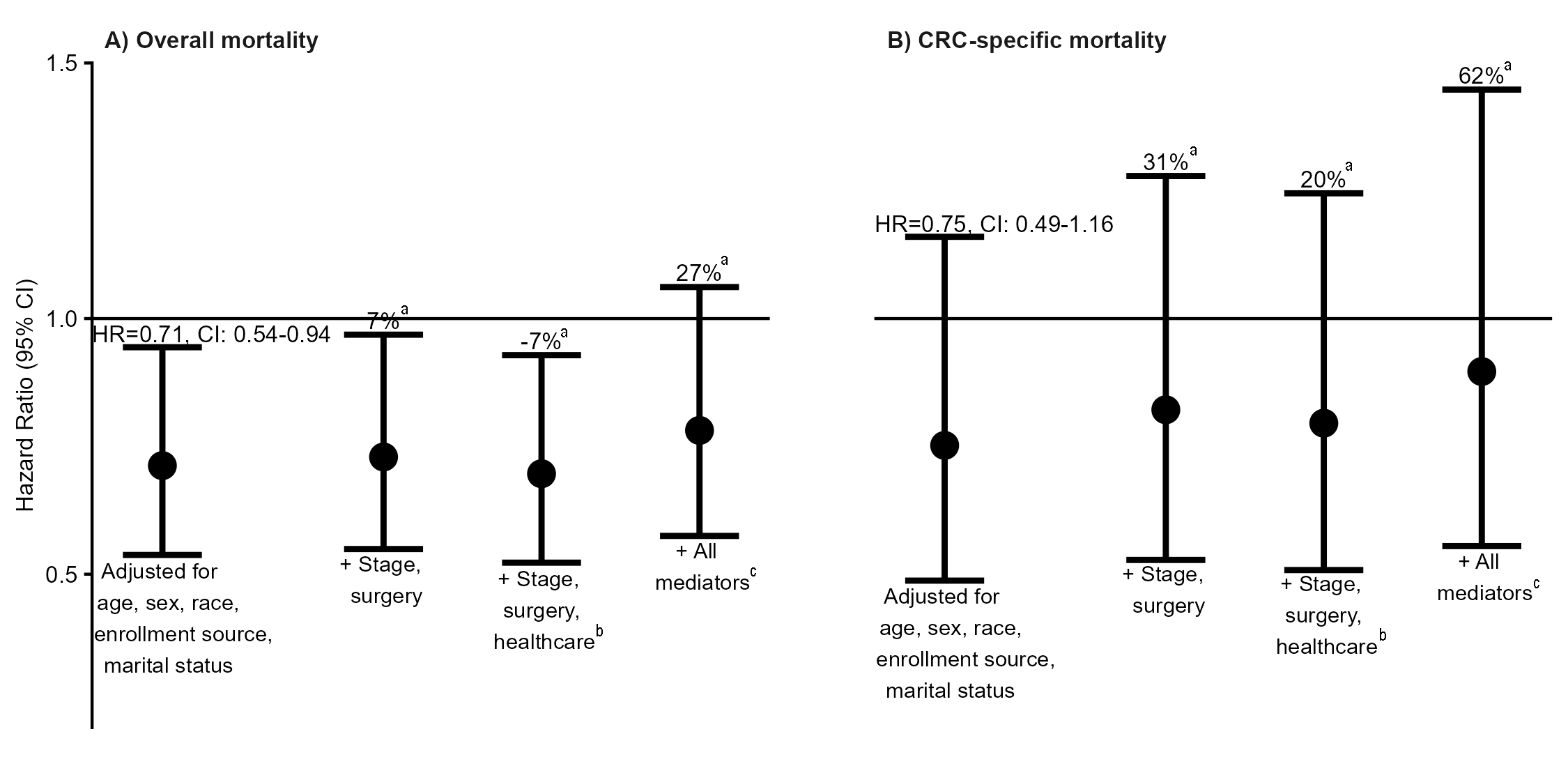


Abbreviations: CI – confidence interval; CRC – colorectal cancer; HEI – Healthy Eating Index

^a^ Percentages reflect the indirect effect of the mediating variables, i.e. the proportion of the log hazard ratio for income that is explained by adjustment for the mediating variables.

^b^ Further adjusted for healthcare-related mediators include screening for colorectal cancer, insurance status at enrollment, and surgical resection of the primary tumor.

^c^ Further adjusted for healthcare-related mediators (see b) and for lifestyle-related mediators including duration of diabetes, smoking status, alcohol consumption, Healthy Eating Index score, and physical activity.
